# Unemployment and labour market recovery policies

**DOI:** 10.1007/s41775-022-00136-x

**Published:** 2022-06-27

**Authors:** Swati Dhingra, Fjolla Kondirolli

**Affiliations:** 1 Centre for Economic Performance and Department of Economics, London School of Economics, London, UK; 2 Centre for Economic Performance, London School of Economics and Department of Economics, University of Sussex, Brighton, UK

**Keywords:** Long-term unemployment, Informal economy, COVID-19 pandemic, Household survey data, E24, E26

## Abstract

Evidence shows long-term unemployment (LTU) can have life-long scarring impacts on the future employment and earning prospects of individuals and lead to an overall deterioration in the wellbeing of communities. This article examines long-term unemployment in India, providing some of the first estimates from a panel of individuals before and during the pandemic. It shows that LTU makes up a substantial proportion of unemployment among the working-age population, particularly among young workers who have fared even worse since the pandemic. Existing benefits have proven inadequate in addressing long-term unemployment and young workers have a strong desire for active labour market policies from the government to address the worklessness crisis. A national-level commitment to active labour market policies could prevent a lost generation of young workers from falling into long-term unemployment and the ills that accompany it.

## Introduction

The pandemic has exacerbated livelihood insecurity among workers across the developing world. Even as the aggregate economy has recovered in many countries since the pandemic, millions of individuals are still experiencing livelihood losses and are facing the risks of long-term unemployment.

Long-term unemployment (LTU) has been a concern in developed countries since at least the eighties after the oil crisis and more recently, after the great recession of 2008 and the huge economic shock from the COVID-19 pandemic. Employment rates fell during these crises, and many of the displaced workers were pushed onto a trajectory of long periods of worklessness. Evidence shows prolonged unemployment has adverse consequences over and above the income and consumption losses from becoming unemployed. Long-term worklessness can have life-long scarring impacts on the future employment and earning prospects of individuals and lead to an overall deterioration in the wellbeing of communities.

The likelihood of an unemployed individual finding a job decreases with time, translating into slower economic growth and structural unemployment. At an individual level, long-term unemployment can discourage individuals from seeking work and therefore induce them to drop out of the labour force altogether. In the long-term, it can lower reemployment wages as workers' reservation wages decline, erode human and social capital, and result in worse physical and mental health for individuals and their communities.^1^Scars from entering a weak labour market and from unemployment spells when young are not transitory, and active labour policies are an important tool to prevent young workers from prolonged worklessness (Arulampalam et al., 2001; Machin & Manning, 1999; Von Wachter, 2020).

Developing economies are characterised by large informal sectors, where workers lack social protection.^2^ High levels of informality are associated with higher levels of poverty and inequality and slower progress toward achieving Sustainable Development Goals. The pandemic has exacerbated livelihood insecurity, especially among informal workers who were more likely to lose their jobs and to be pushed into poverty (Ohnsorge & Yu, 2021). Even as the aggregate economy has recovered in many countries since the pandemic, millions of informal workers are still experiencing livelihood losses and are facing the risks of long-term unemployment.

India has a large informal workforce, typical of developing countries,^3^ and it also suffered one of the deepest economic contractions from the pandemic (see Ray & Subramanian, 2020). High unemployment was a feature of the labour market even before the pandemic (Deshpande & Singh, 2021; Gupta & Kishore, 2021), and the pandemic caused sharp increases in unemployment, especially among young workers in low-income urban areas, which were at the frontlines of the pandemic (Bhalotia et al., 2020; Dhingra & Kondirolli, 2021, see Azim Premji University, 2021). While GDP has recovered to its pre-pandemic levels since 2021, unemployment has remained above its pre-pandemic levels, especially among young workers. At the time of writing this article (April 2022), unemployment was termed the "biggest challenge" for India in the light of the pandemic and the demographic transition to a higher share of working-age individuals in the population.^4^

For demographic transitions to provide growth dividends, young workers need to be gainfully employed. But many developing economies, including India, face a jobs crisis, which has been made more severe by the pandemic. This article examines unemployment in India, providing some of the first estimates of long-term unemployment (LTU) from a panel of individuals before and during the pandemic. It shows that LTU makes up a substantial proportion of unemployment among the working-age population, particularly young workers who have fared even worse since the pandemic. It then examines the potential of different labour market recovery policies in delivering a transformative recovery from long-term worklessness in India and, more broadly, in developing economies where the majority of the workforce is young and informally employed.

## Long-term unemployment

While long-term unemployment is a perennial area of research and policy in the developed world, it has received less attention in the context of emerging markets and developing economies. Data shortcomings in the developing world make it difficult to shed light on this problem, despite the big challenges it raises for economic policy and the wellbeing of young individuals who dominate the labour force. Panel labour force surveys are scarce, and administrative data typically does not capture the informal economy fully. Consequently, there is a limited understanding of the extent and incidence of LTU. Using the panel structure of the Consumer Pyramids Household Survey (CPHS) data of the Centre for Monitoring the Indian Economy (CMIE), this section examines the evolution of LTU at a national level and among the youth population.

We present findings from a panel of individuals whose employment status and unemployment durations are obtained from the CPHS between January 2017 to August 2021. CPHS is a panel survey conducted three times per year, and the employment status of an individual is recorded during each of those three times. Individuals report their employment status (employed, unemployed and looking for work, unemployed and not looking for work, or unemployed and out of the labour force).

*Unemployment ranges from about 7–10%, and on average, unemployed individuals are out of work for nine months. Urban areas have higher unemployment and unemployment duration than rural areas, but rural areas also face both. Youth unemployment is four times the national rate, and the young are, on average, unemployed for over one month longer than the national average.*

Unemployment is recorded based on daily recall at the time of the interview, and the unemployment rate is defined as the share of unemployed individuals in the labour force (i.e. those who are employed or unemployed and looking for work). Overall, the unemployment rate during the period was 7% of the labour force (Table 1).

**Table 1:** Unemployment and long-term unemployment in India, 2017–2021

	National	Rural	Urban	Younger 15–25 years	Older > 25 years
Unemployed	7.06%	6.67%	7.92%	29.63%	2.24%
Unemployment duration (months)	9.03	8.63	9.74	10.15	5.86
Unemployed for					
< 3 months	45.55%	46.68%	43.57%	39.70%	62.13%
≥ 3– < 6 months	14.45%	14.38%	14.59%	15.16%	12.45%
≥ 6– < 12 months	9.54%	9.67%	9.35%	10.70%	6.28%
≥ 12– < 24 months	16.13%	15.83%	16.67%	18.00%	10.98%
≥ 24 months	14.31%	13.45%	15.82%	16.48%	8.16%
Share of labour force	100%	67.78%	32.22%	17.62%	82.38%
Observations	2,436,038	876,432	1,559,606	442,517	1,993,521

Notes: Source: Consumer Pyramids Household Survey. Sample includes individuals 15 to 64 years old in the labour force. In labour force includes individuals who were employed or were unemployed and looking for a job. *Unemployed* is the share of individuals in the labour force who were unemployed and looking for a job. *Unemployment duration* is the calculated number of months an individual has been unemployed during the ongoing spell of unemployment in the period. It is calculated from the observed employment status during the interview in each round of the survey and from the reported unemployment duration for the first-ever round of interviews of the individual and for individuals that switch from employment to unemployment in subsequent interviews. *Unemployed for* is the share of the unemployed that are unemployed for the stated duration

**Fig. 1:**
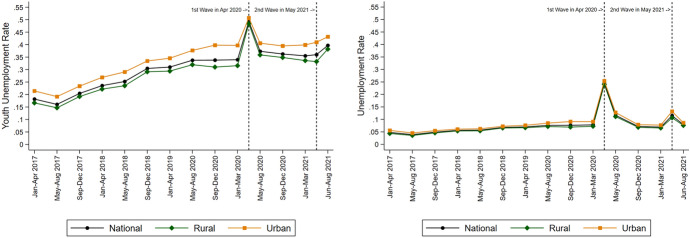
Unemployment and Youth Unemployment Evolution in India, 2017–2021

Unemployment started at about 6% before the pandemic, between Jan 2017 to Dec 2019. It jumped to 24.6% in April 2020 during the peak of the first wave of the pandemic when India was under a strict lockdown and data collection suffered as well. The unemployment rate returned to about 8.3% afterwards, excluding May 2021, when it was 11.5% due to the second wave of the pandemic. Despite a period of economic recovery, unemployment hovered at a higher level than before the pandemic (Fig. 1, left panel).

**Fig. 2:**
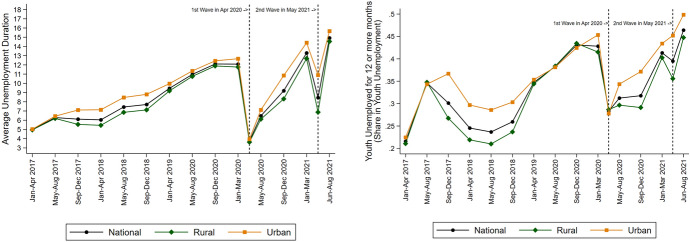
Unemployment and Youth Unemployment Duration in India, 2017–2021

Unemployment duration is the number of months that the individual has been unemployed and looking for work during the ongoing unemployment spell in each of three rounds in the year. The main unemployment length measure is based on employment status and employment duration reported every four months.^5^ On average, unemployed individuals are unemployed for nine months (Table 1; Fig. 2, left panel).

Urban areas have slightly higher unemployment rates and longer unemployment duration than rural areas (Table 1; Figs. 1, 2 left panels). They have also faced higher unemployment since the pandemic. Yet unemployment is not just an urban phenomenon. Rural areas also have substantial unemployment and unemployment durations and have seen a sharp fallout from the pandemic. The rural–urban comparisons come with the caveat that the CPHS data has a better representation of urban areas than rural areas, as noted in various studies (Dhingra & Ghatak, 2021; Drèze & Somanchi, 2021).^6^

What is striking is the particularly high unemployment among young workers between 15 to 25 years.^7^ Young workers make up the bulk of unemployed individuals, and they are also expected to face a much higher burden of the scarring effects of prolonged periods of unemployment through livelihood losses, lower future earnings, reduced human capital accumulation and well-being, and potential criminal activities. About 30% of this age group are unemployed and face an average unemployment duration of over ten months, slightly less than double those of older workers. They have also fared particularly badly since the pandemic, with unemployment rates soaring to about half during the peak of the first wave of the pandemic. While this quickly came down, it settled 10% points higher than the pre-pandemic unemployment rate of 26%.

*Long-term unemployment often measured as unemployment of a year or more, makes up about a quarter of unemployment. It is about 3 pp higher in urban areas compared to rural areas. It is primarily driven by long-term unemployment among the youth.*

About 40% of unemployed individuals have been without a job for more than half the year, with 16 and 14% facing unemployment for 1–2 years and over two years, respectively. The only period during which this falls sharply is the peak of the 2020 lockdown when the share of unemployed individuals in long-term unemployment naturally fell as more people entered unemployment during the pandemic. Urban areas have higher long-term unemployment, with over a third of unemployed workers being out of work for a year or more.

Unemployment and particularly long-term unemployment is driven by younger individuals. About 35% of unemployed youth have been unemployed for more than a year, compared to 19% of older workers. The youth make up about 70% of unemployed individuals and a much higher 84% of the long-term unemployed. The likelihood of scarring effects is substantial as a large proportion of unemployed youth have already entered long-term unemployment, particularly in urban areas where they have also suffered more from the labour market losses since the pandemic.

**Fig. 3:**
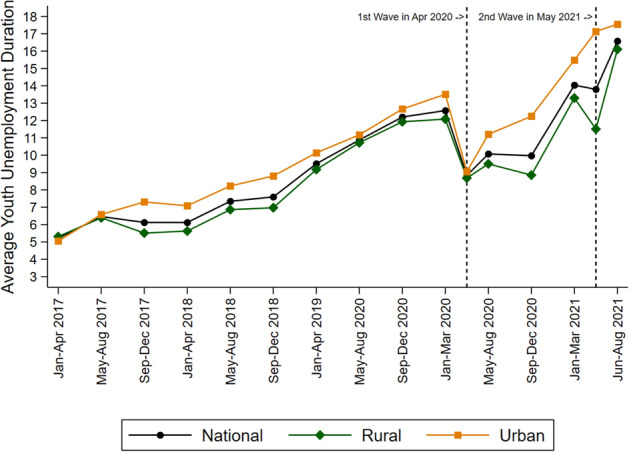
Long-term youth unemployment (≥ 12 months) in India, 2017–2021

The problem of long-term youth unemployment is more acute in urban areas and has become much worse since the pandemic (Fig. 3). Still, rural areas also show an alarmingly high youth unemployment rate of a third, despite the availability of agriculture and the rural employment guarantee, which has provided a lifeline for displaced workers through the pandemic.

Overall, long-term unemployment, typically measured as spells of 12 months or longer, makes up a substantial proportion of unemployment in India, and the burden falls disproportionately on younger workers. To put these numbers in perspective, the share of LTU of 12 months or more was 12–15% in the United States between 2017 and 2019 and about 6% in 2020. As European Union countries have higher LTU rates, the comparable figures for G7 countries were 28–30% and 17%, respectively. Few emerging and developing economies have data on LTU shares, but among those that are available, the shares in Mexico, Colombia and Namibia are low (< 2, < 10 and < 15%, respectively), while Turkey and Russia are in the range of 20–25% and 20–30% respectively.^8^

Youth unemployment rates are higher in India compared to any of the emerging or developing economies in the OECD database during this period. Yet many other countries implemented wider labour market policies than India to prevent the scarring effects of prolonged unemployment for their young workforces. Worldwide, an astonishing 1300 different jobs programmes have been adopted to rebuild the livelihoods of millions of informal workers and prevent them from long-term unemployment (Khamis et al., 2021). The following section presents some evidence for various job market policies that have been discussed to address the burgeoning jobs crisis in India and worldwide.

## Labour market policy

Research on the impact of active labour market policies (ALMPs) such as training, job-search assistance, subsidised private and public employment, or a combination of the above shows that these policies have the potential to effectively address long-term unemployment even after periods of economic crises. Specifically, they are more effective in addressing long-term unemployment, such as through human capital formation and training, which are usually not the focus of policies designed for tackling short-term unemployment.

Active labour market policies have seen renewed interest across the world. India already runs the world’s largest job guarantee programme under its Mahatma Gandhi National Rural Employment Guarantee Act (MGNREGA), which entitles rural households to demand 100 days of work a year from the government. Demand for work under the programme went up by 1 billion person-days of work after the pandemic.^9^ Proposals to address the deep unemployment crisis in urban areas include policies such as an urban job guarantee to expand the remit of the existing rural job guarantee programme (Azim Premji University, 2019, 2021), a Decentralised Urban Employment and Training programme (Drèze, 2020) and a multi-year paid government internship programme (Banerjee et al. 2019). They are expected to fill the gap created by the private sector's continued inability to generate decent work for the large young informal workforce of the country.

A large literature seeks to evaluate the performance of the national rural employment guarantee and finds positive impacts on wages, livelihoods, and the creation of public assets. While these can be studied from ex-post evidence of the programme, many active labour market policies remain untried, and there is a paucity of evidence on what policies might be effective in urban settings and in addressing youth unemployment. To fill this gap, we report findings from a primary survey in low-income urban areas that was designed to examine which policies are reaching workers who have experienced livelihood losses from the pandemic and which policies they expect would be most effective in addressing unemployment in their areas.

The primary survey, conducted by the LSE’s Centre for Economic Performance (CEP), collected information from a random sample of individuals from the low-income states of Bihar, Jharkhand and Uttar Pradesh. These three states have patterns of evolution and durations of unemployment and youth unemployment that are similar to the rest of the country. The youth unemployment impacts that they incurred from the pandemic were slightly higher than the national average and the unemployment durations were slightly shorter. Individuals who had worked before the pandemic and were between 18 to 40 years old were interviewed to understand the experience of individuals who have been in the labour force and whose work may have been impacted by the pandemic.

*Existing labour market benefits programmes are reaching a small share of urban workers.*

The survey covered information on a number of benefits: Provident fund, Pension, Paid Sick Leave, Account with Employee's Provident Fund Organization (EPFO), Account with Employee's State Insurance Corporation (ESIC), or Central Government Health Scheme, provided by the government or employers to protect workers from livelihood insecurity. Seventy-three per cent of workers have none of these benefits. Those employed informally and those belonging to lower socioeconomic groups are even less likely to have any job protections. Just 15% of informally employed workers have some form of benefits, compared to 82% of formal workers or 83% of regular salaried workers.

These numbers mirror the Periodic Labour Force Survey results, which show that less than half of workers have regular salaried employment, and even among them, a majority lack access to any non-wage benefits such as social security, pension and sick leave. Moreover, the government's flagship programmes, such as ESIC and EPFO, were designed to provide social protection to informal workers, but their coverage remains low. For example, although the government expanded the EPFO during the pandemic, its website shows that the scheme reportedly covers just 63 million of the nearly half-billion individuals in the labour force. Therefore, the existing benefit system falls far short of the universal coverage and the scale of the informal workforce.

*Urban workers strongly support public programmes to address unemployment, with job guarantees being the top choice of the overwhelming majority, followed by cash transfers.*

The survey asked about opinions on different labour market policies to tackle unemployment. To reduce framing bias, questions on labour market policies were framed differently, and individuals were randomly assigned to the questions (see Dhingra & Kondirolli, 2021 for details).

Respondents were asked to choose between various policies paid for by the government that they thought would be most effective in tackling unemployment in urban areas. The various policy options included job guarantees for urban workers, direct cash transfers for urban individuals, wage subsidy to reduce labour costs for industry in the area, land grants, tax holidays or other incentives to the industry in the area, and an open-ended other option. Eighty-two per cent of the respondents say that job guarantee programs would be the most effective in solving the problem of unemployment in urban areas, followed by cash transfers (16%), wage subsidies (1%), land grants and tax holidays (1%), and others (0.1%).

The survey also asked all respondents their preference between the top two options of a job guarantee and a cash transfer. Respondents were first asked how good, on a scale of 0–10, they think each policy, paid by the government, would be in tackling unemployment in urban areas. They were then asked which of the two options they would prefer in their area. The order of appearance of each option was randomly assigned, and there were no systematic differences in the policy choices of workers by order of the options they received. When choosing between a job guarantee and a cash transfer, 84.5% prefer a job guarantee. While 13% of the sample had received cash transfers since the pandemic, even among these recipients, about 78.5% prefer a job guarantee over a cash transfer.

The proportion of employed and unemployed individuals choosing job guarantees or cash transfers and their reasons for doing so are given in Table 2. The vast majority—86% overall—of those who chose a job guarantee over a cash transfer said that job guarantees would directly address the lack of work or livelihood insecurity. Other reasons include certainty of government payments, local work opportunities, and more days of work.

**Table 2:** Labour market policy preferences of urban workers by employment status

	Employed	Unemployed
Prefer a job guarantee over a cash transfer	0.87	0.81
Why, if prefer job guarantee? Job guarantee will directly address the lack of work or directly address livelihood insecurity	0.86	0.86
Workers are sure to get paid from the government, even if there are delays	0.03	0.10
People need work in their areas	0.05	0.02
People need more days of work	0.04	0.02
Prefer cash transfer over a job guarantee	0.13	0.19
Why, if prefer cash transfer?		
Cash transfers are more flexible	0.43	0.09
Wages under job guarantees are too low	0.07	0.30
Job guarantees are run by job contractors	0.06	0.20
Job guarantee work is too rationed	0.11	0.12
Cash transfers will enable people to do or look for better work	0.08	0.04
Sample size	2962	1801

Notes: Source: LSE-CEP Survey. Preferred policy refers to the policy respondents think would most effectively tackle unemployment in urban areas. Unemployed refers to individuals who were unemployed the week before the survey, which includes those who were unemployed and looking/not looking for work but who had employment before the pandemic

Among those who prefer a cash transfer, the main reason is the flexibility that cash provides (28% overall). Seventeen per cent said that job guarantee wages are too low. The Government of India has made paltry increases in wages under the rural job guarantee, and there have also been concerns over job rationing in the past. This seems to be reflected in some individuals preferring cash transfers due to the low earning potential of job guarantees from low wages and rationing. The role of contractors in job guarantees also contributes to some individuals preferring a cash transfer.

In sum, both employed and unemployed workers have a frustrated demand for active labour market policies, particularly job guarantees to address worklessness and livelihood security.

## Conclusion

The evidence in this article shows that long-term unemployment has been a structural feature of the Indian labour market in recent years. Existing active labour market policies have proven inadequate and would need a substantial increase in scope and depth to help alleviate the worklessness crisis that is unravelling in the country. A national-level commitment with substantial resource allocation, which is otherwise out of the reach of many state governments, would be needed to achieve this.

A serious debate over various policy options, their trade-offs and their potential impacts on long-term unemployment would be an important first step towards designing social safety nets, which continue to be out of the reach of many workers. India has experience with innovative labour market policies, which provides important and rare learning opportunities for future labour market initiatives. They will also provide an invaluable resource for evidence to inform research and policy now and in the future, and across the developing world, where informality and youth unemployment are massive challenges.

India already runs the world’s largest job guarantee programme in rural areas and proposals to formulate an employment guarantee in urban areas range from wage subsidies for employers to direct employment by public institutions (Drèze, 2020; Kulkarni & Ambasta, 2020). There is a dearth of knowledge, however, on how to operationalise these policies in urban labour markets where individuals have much more varied skill levels than agrarian economies. Urban areas also differ in that they lack the tight community ties or local governance structures of villages, where various monitoring mechanisms have contributed to individuals actually receiving their work entitlements (Drèze & Khera, 2009).

A few state governments have introduced an urban equivalent of MGNREGA, though budgets are relatively small (Azim Premji University, 2021). The central government has indicated plans for an urban job guarantee in small towns and cities to address the crisis, but no new policy is yet in place (see Dhingra & Machin, 2021). This is in stark contrast to the rest of the world where a number of public employment programmes and relief packages for informal workers have been put forward under COVID-19, such as COVID-safe extensions to South Africa’s Expanded Public Works Programme.^10^ International organisations, such as the OECD and the ILO, have explicitly called for job guarantees to prevent a permanent deterioration in work and living standards for young workers (ILO, 2020; OECD, 2020).

Young workers have a strong demand for policies to create job opportunities. They have already witnessed decades of inadequate work. They have now gone through the worst economic crisis in modern India and are far from fully recovering to even the weak pre-pandemic labour market. Implementing active labour market policies can prevent another lost generation of young workers from falling into the despair of worklessness and the ills that accompany it. Active labour market policies offer a ray of hope in ensuring that the demographic dividend is not squandered away.

## Footnotes

1 See, for example: Jacobson et al. (1993), Ruhm (1991), Sullivan and von Wachter (2009), Browning and Heinesen (2012), Eliason and Storrie (2009), and Bentolila and Jansen (2016) for LTU from the pandemic.

2 In emerging markets and developing economies, informality makes up one-third of economic output and two-thirds of employment, with employment shares as high as about 90% in Mali, Mozambique, and India (Ohnsorge & Yu, 2021).

3 In 2018, informal employment amounted to 88.6% of total employment in India, with similar rates in the region (81% in Nepal, 94.7% in Bangladesh, 81.7 in Pakistan), but higher rates than Latin American countries (69.4 Peru, 62.4 Colombia) and much higher rate than for example South Africa 35.3% (Ohnsorge & Yu, 2021).

4 https://indianexpress.com/article/opinion/editorials/unemployed-indians-biggest-challenge-7890582/.

5 The earliest employment status is available from January 2016 so we report numbers from January 2017 onwards when long-term unemployment of a year or more can be observed. Unemployment duration is calculated as the number of months between two consecutive unemployment observations when the reported unemployment duration is not lower than the period of the consecutive observations and the reported duration otherwise. We do not account for the unemployment duration of an individual reported in the month of initial entry into the panel because this is often based on much longer recall periods that differ from the 4-month periodicity of the survey. Nonetheless broad patterns are highly similar to those in the article when the initial duration is also added to the unemployment duration, though the magnitudes of unemployment durations are much larger because of many high initial duration values which could be reflecting a more severe chronic unemployment problem. Self-reported measures of unemployment length from cross-sectional questions that ask respondents their duration of unemployment in each round, show much longer durations of unemployment, over 50 months. This could be due to differences in recall periods or chronic unemployment. Yet, broad patterns reported in this article are similar when using the self-reported durations.

6 Moreover, the CPHS data collection during the first wave of the pandemic in India was limited as the household interviews switched from in-person to phone surveys. The response rate decreased from an average of 85% pre-COVID to 35% during the first wave.

7 Results are broadly similar when the age range is altered to 18–25 to account for youth of school-going ages.

8 https://data.oecd.org/unemp/long-term-unemployment-rate.htm. The remaining developing economy in the OECD database is South Africa, which reports high rates of 60 percent and compares well with the self-reported months in the CPHS and National Sample Survey Organisation cross-sectional data (see Ahmed, 2015; Naraparaju, 2017).

9 https://timesofindia.indiatimes.com/india/after-2020-21-record-peak-dip-in-rural-jobs-scheme-demand/articleshow/90602341.cms.

10 New and expanded job guarantee programmes have been put in place in various countries, including Armenia, Azerbaijan, Bhutan, Cambodia, Ethiopia, Grenada, Guam, Guinea, Hong Kong, India, Indonesia, Kazakhstan, Kenya, Korea, Madagascar, Mexico, Myanmar, Nepal, Nigeria, Palau, Philippines, Rwanda, St Lucia, St Vincent and Grenadines, Taiwan, Uganda, Uzbekistan, West Bank and Gaza, and Zimbabwe.
